# Immunosuppressive effect of PLGA-FK506-NPs in treatment of acute cardiac rejection via topical subcutaneous injection

**DOI:** 10.1080/10717544.2021.1968978

**Published:** 2021-08-31

**Authors:** Cheng Deng, Qiaofeng Jin, Ya Wu, Huiling Li, Luyang Yi, Yihan Chen, Tang Gao, Wenyuan Wang, Jing Wang, Qing Lv, Yali Yang, Jia Xu, Wenpei Fu, Li Zhang, Mingxing Xie

**Affiliations:** a Department of Ultrasound Medicine, Union Hospital, Tongji Medical College, Huazhong University of Science and Technology, Wuhan, China; b Hubei Province Key Laboratory of Molecular Imaging, Wuhan, China

**Keywords:** Heart transplantation, acute rejection, administration route, subcutaneous drug delivery, nanoparticles

## Abstract

FK506, a first-line immunosuppressant, is routinely administered orally and intravenously to inhibit activation and proliferation of T cells after heart transplantation (HT). Current administration route is not conducive enough to exert its efficacy in lymphatic system. Herein, we proposed that subcutaneous (SC) administration of FK506-loaded nanoparticles (PLGA-FK506-NPs) would be valuable for treating acute rejection after HT. The biodistribution and pharmacokinetic study revealed that it could effectively deliver FK506 to the lymph nodes (LNs) due to their suitable particle size, especially in inguinal LNs. Subsequently, the therapeutic efficacy of PLGA-FK506-NPs on the HT model was evaluated using intravenous (IV), intragastric (IG), or SC injection. Histopathological analysis revealed that 80% of allografts exhibited only grade 1R rejection with negligible lymphocyte infiltration in the SC group. The IV group exhibited 40% 1R rejection with mild lymphocyte infiltration and 20% grade 3R that require further intervention, and the IG group exhibited grades 40% 3R rejection with more lymphocyte infiltration. Moreover, the infiltration of T cells and the secretion of IL-2 and IFN-γ were significantly reduced in the SC group compared with the IG or IV group. The mean survival time (MST) further revealed that 50% of grafts treated with PLGA-FK506-NPs via SC injection survived longer than IG and IV groups. Moreover, the MST of single-dose SC injection of PLGA-FK506-NPs demonstrated that it would effectively reduce the required dose for a similar therapeutic effect. Overall, these results indicate that SC administration of PLGA-FK506-NPs is a more effective route for chronic FK506 treatment.

## 1. Introduction

Heart transplantation (HT) is a standard treatment for patients with irreversible end-stage heart failure, and acute rejection is one of the major causes of morbidity and mortality after HT (Olymbios et al., 2018; Stehlik et al., 2018). Patients have to take immunosuppressants for the rest of their life to prevent and treat the occurrence of acute rejection. Currently, tacrolimus (FK506), which inhibits calcineurin by binding with FK506-binding protein to suppress various cytokines production and T cell proliferation and activation, has been widely used to treat solid organ transplantation rejection and many other immunological disorders (Schreiber & Crabtree, 1992). However, long-term use of FK506 brings huge economic costs and inevitably causes many side effects, including nephrotoxicity, opportunistic infections, and malignancies. Many studies have developed FK506 delivery systems to prolong survival time and reduce side effects, and some of the new strategies have been widely studied in skin, liver, corneal, and islet transplantation (Xu et al., 2014; Shahzad et al., 2018; Tung Thanh et al., 2018; Wu et al., 2019; Xie et al., 2020).

It is well-known that the lymphatic system, such as lymph nodes (LNs) and spleen, is a primary site for FK506 to inhibit the priming and activation of T lymphatic cells (Azzi et al., 2016; Wu et al., 2020). Generally, FK506 is routinely administered orally once or twice a day, and intravenous (IV) administration is an alternative route in patients for early treatment after transplantation or when the oral route is not available (Zamorano-Leon et al., 2016). However, it has been demonstrated that only a small amount of FK506 enters the lymphatic system by IV or oral administration (Khan et al., 2016; Yoshida et al., 2016). Therefore, optimizing treatment administration and improving curative effect is urgent and necessary. In addition, many studies have confirmed that the route of administration influences the bioavailability, absorption, distribution, and clearance of drugs (Mohanan et al., 2010; Pennington & Park, 2015; Dogra et al., 2018; Niu et al., 2019). In the case of oral administration, absorption of FK506 in the gastrointestinal tract may be affected by variability in drug absorption/metabolism, resulted in poor bioavailability, high pharmacokinetic variability. However, seldom efforts have been paid to find a new alternative administration route to improve their bioavailability for chronic FK506 treatment.

Subcutaneous (SC) delivery of biotherapeutics has shown to be effective, safe, and well-tolerated, and it has become a valuable alternative to IV administration across many disease areas (Bittner et al., 2018; Collins et al., 2020). Following SC administration, small molecules, peptides, and small proteins (≤16 kDa) primarily diffuse directly into capillaries through the blood vessel walls, whereas large molecules are taken up into the more porous lymphatics (McDonald et al., 2010). However, the FK506 has a small molecular weight of less than 1000 and a hydrophobic character, which is not available for SC administration of FK506 directly. Therefore, a suitable carrier would provide the solution. Biodegradable polymers have been widely studied over the past two decades to improve solubility and bioavailability (Singh & Lillard, 2009). Accordingly, a large number of studies have confirmed that poly(lactide-co-glycolide) (PLGA) is a biocompatible, biodegradable, nontoxic, and non-immunogenicity polymer widely used in designing microparticles or nanoparticles (Danhier et al., 2012; Mitragotri et al., 2014; Kalam & Alshamsan, 2017; Martins et al., 2018; Alshamsan et al., 2020). Our previous study suggests that PLGA-FK506-NPs is a safe and efficacious formulation with a prolonged release property that can be used to treat acute rejection without significant adverse effects (Deng et al., 2020). Herein, we hypothesized that SC administration of PLGA-FK506-NPs would result in improved therapeutic efficacy.

In this study, we characterized the physicochemical properties of PLGA-FK506-NPs. We further studied the biodistribution of DiR-labeled-NPs following various routes of administration by using a small animal imaging device. The pharmacokinetics of PLGA-FK506-NPs with different administration routes were performed using a high-performance liquid chromatography–mass spectroscopy (HPLC–MS) system. Finally, the effect of the PLGA-FK506-NPs on cardiac graft survival and cytokine secretion with different administration routes were studied using a rat heterotopic HT model.

## 2. Materials and methods

### 2.1. Materials

PLGA (lactide:glycolide ratio 50:50, molecular weight = 30,000–60,000) was purchased from Sigma-Aldrich (St. Louis, MO). FK506 was obtained from MCE (Princeton, NJ) with purity >99.93% (LCMS). DiR (1,1′-dioctadecyl-3,3,3′,3′-tetramethylindotricarbocyanine iodide) was obtained from AAT Bioquest (Sunnyvale, CA). All the other chemicals used in this study were of analytical grade.

### 2.2. Animals

Male Sprague-Dawley (SD, 200–250 g), Brown Norway (BN, 200–250 g), and Lewis (200–250 g) rats were purchased from Vital River Laboratory (Beijing, China). All animal procedures were performed following the Guidelines for Care and Use of Laboratory Animals of Huazhong University of Science and Technology and approved by the Animal Ethics Committee of Tongji Medical College, HUST.

### 2.3. Preparation of PLGA-FK506-NPs

According to our previously established method, PLGA-FK506-NPs were prepared by the O/W emulsion solvent evaporation method (Deng et al., 2020). More specifically, FK506 (1 mg) and PLGA (16 mg) were dissolved in a 0.5 mL mixture of ethyl acetate and dichloromethane (1:1). The mixture was added to an aqueous phase containing a surfactant (F-68, 1.25%w/v, 2 mL) and was sonicated with a probe sonicator for 5 min (LC1000N, Ultrasonic processor, Ningbo, China) to generate an oil-in-water emulsion. The resultant emulsion was diluted to 10 mL with F-68 solution (0.5% w/v), and the solvents were removed using a rotary vacuum evaporator (RE-52A, Yarong Biochemistry, Shanghai, China) for 15 min. The resultant dispersion was centrifuged for 15 min at 4000 rpm using an amicon ultra-4 centrifugal filter (MWCO: 10k, Millipore, Billerica, MA) to remove the free drug. Finally, the residue was collected and lyophilized using trehalose (10% w/v) as a cryoprotectant. DiR-labeled PLGA-NPs were prepared in the same method by adding 50 µL DiR (1 mg/mL) to the initial organic mixture.

### 2.4. Characterization of PLGA-FK506-NPs

Size distribution, zeta potential, and polydispersity of PLGA-FK506-NPs were performed by dynamic light scattering (DLS) using a zeta potential analyzer (ZetaPALS, Brookhaven Instruments, Holtsville, NY). The morphologies of PLGA-FK506-NPs were determined by transmission electron microscopy (TEM, Hitachi HT7700, Tokyo, Japan) and scanning electron microscopy (SEM, Hitachi SU8010, Tokyo, Japan). The entrapment efficiency (EE) and drug loading efficiency (LE) were determined by high-performance liquid chromatography (HPLC) with a C18 column (4.6 mm × 250 mm, 5 µm) at 40 °C, and a mixture of acetonitrile/0.1% phosphoric acid solution in water (70/30, v/v) was adopted as mobile phase (Tung Thanh et al., 2018). The flow rate was 1 mL/min. The FK506 was detected at a wavelength of 210 nm. EE and LE were calculated as follows: EE (%)=(weight of FK506 in nanoparticles/weight of FK506 added)×100; LE (%)=(weight of FK506 in nanoparticles/weight of nanoparticles)×100. The physical characterization of PLGA-FK506-NPs was determined by Fourier transform infrared (FTIR) spectrometry using a PerkinElmer FT-IR spectrometer, as described previously (Pathak et al., 2017). *In vitro* release tests were conducted in 1× phosphate buffer saline (pH 7.4, mixed with 0.5% Tween-80) using a dialysis method (Zhao et al., 2015), and the FK506 was measured by the HPLC method described above. All measurements were repeated in triplicate.

### 2.5. Biodistribution study

SD rats were used to study the biodistribution of NPs via IV, intragastric (IG) or SC administration of DiR labeled PLGA-NPs. Trafficking of the DiR labeled PLGA-NPs was evaluated using a small animal imaging system (In-Vivo FX PRO, BRUKER, Billerica, MA) equipped with a 750 nm excitation filter and a 790 nm emission filter. The hearts, livers, spleens, lungs, kidneys, mesenteric lymph nodes (MLNs), inguinal lymph nodes (ILNs) and axillary lymph nodes (ALNs) were harvested and analyzed for the biodistribution 24 h post-injection.

### 2.6. Pharmacokinetics study

To compare the pharmacokinetic profile of PLGA-FK506-NPs with different routes of administration, 18 SD rats were administrated with the same dose (1 mg/kg) by IV, SC, or IG routes (*n* = 6). Blood samples were collected from the jugular vein at predetermined time intervals (0.25 h, 0.5 h, 1 h, 2 h, 4 h, 8 h, 12 h, and 24 h). To evaluate the concentration of FK506 in spleen and LNs, the spleen, MLNs, ILNs, and ALNs were then isolated and weighed 24 h after various administrations. The concentration of FK506 in whole blood and tissues was measured by an HPLC–MS system (UltiMate 3000 RS and TSQ Quantum, Thermo Fisher Scientific Inc., Waltham, MA) using a previously reported method with some modifications (Shin et al., 2010; Deng et al., 2020). Briefly, a mixture of 100 µL of whole-blood sample and 300 µL acetonitrile was vortexed and centrifuged at 15,000 rpm for10 min. After that, 10 µL of cyclosporin A (2 µg/mL) was added into the 100 µL supernatant as an internal standard. Then, the samples were measured in a C18 column (2.1 mm × 100 mm, 1.9 μm) at 35 °C. A mixture of methanol solution and 0.1% formic acid aqueous solution was used as the mobile phases. The flow rate was 0.4 mL/min, and the injection volume was 5 µL. In addition, the tissues were homogenized with 1.0 mL of dichloromethane solution and centrifugated at 14,000 rpm for 10 min. Then, 100 µL of the organic solvent was put into a tube and dried in nitrogen, then the residue was dissolved in 150 µL methanol with 10 µL of cyclosporin A (2 µg/mL), and injected into the HPLC–MS system for analysis. Pharmacokinetic analysis was performed by using the DAS2.0 program, as previously reported (Zhao et al., 2015).

### 2.7. Heterotopic abdominal cardiac transplantation

BN and Lewis rats at 8–10 weeks of age were used as cardiac donors and recipients, respectively. BN hearts were transplanted into Lewis recipients as allografts, and Lewis hearts were transplanted into Lewis recipients as isografts (ISO). Heart transplantation was performed by using a microsurgical technique previously described (Ono & Lindsey, 1969). In brief, the donor heart was harvested, and the ascending aorta and pulmonary artery of the donor heart were sutured to the abdominal aorta and the vena cava of the recipient rat, respectively. To analyze the immunosuppressive response of the PLGA-FK506-NPs, the rat heterotopic cardiac transplantation model was divided into five groups (*n* = 4–6). The PBS group as a negative control and the ISO group as a positive control. The other three groups are PLGA-FK506-NPs solution injected via IV, IG, or SC at a dose of 1 mg/kg on a postoperative day (POD) 1–5. Moreover, another three groups were given 1, 2, or 3 mg/kg single injection on POD 1. The survival time was defined as complete cessation of heart beats evaluated by palpation or transabdominal echocardiography with a follow-up time of 28 days. While different routes of administration were well tolerated, and there was no sign of local infection or other symptoms relating to SC injection during the observation period.

### 2.8. Histological, immunohistochemistry, and immunofluorescence analysis

Allografts were harvested at POD 7 or at the time of complete cessation of heart beats. The tissues were fixed in formalin and embedded in paraffin. The paraffin-embedded tissue sections were stained with hematoxylin and eosin (H&E) staining, and the grade of acute rejection was assessed according to the criteria of ISHLT (Stewart et al., 2005). T lymphocyte infiltration of the tissues was determined by immunohistochemical staining with anti-CD3 antibody (ab5690, Abcam, Cambridge, UK). The secretion of inflammatory cytokines was determined by immunofluorescence with anti-IFN-γ (ab216642, Abcam, Cambridge, UK), anti-IL-2 antibody (ZI091710A, R&D, Minneapolis, MN) and anti-IL-6 (GB11117, Servicebio, Wuhan, China). Image-J software was used to quantify fluorescence images (Bethesda, MD).

### 2.9. Statistical analysis

Statistical analysis was performed using Prism8.0 GraphPad software (GraphPad Software, La Jolla, CA). Results were reported as mean ± standard deviation (SD), *n* = 4-7. Significances of differences were determined using Student’s *t*-test or one-way analysis of variance (ANOVA). Survival of cardiac grafts was examined by Kaplan–Meier’s analysis, and group comparisons were performed using the log-rank test. *p* Values <.05 were considered statistically significant.

## 3. Results

### 3.1. Characterization of PLGA-FK506-NPs

**Figure 1.**
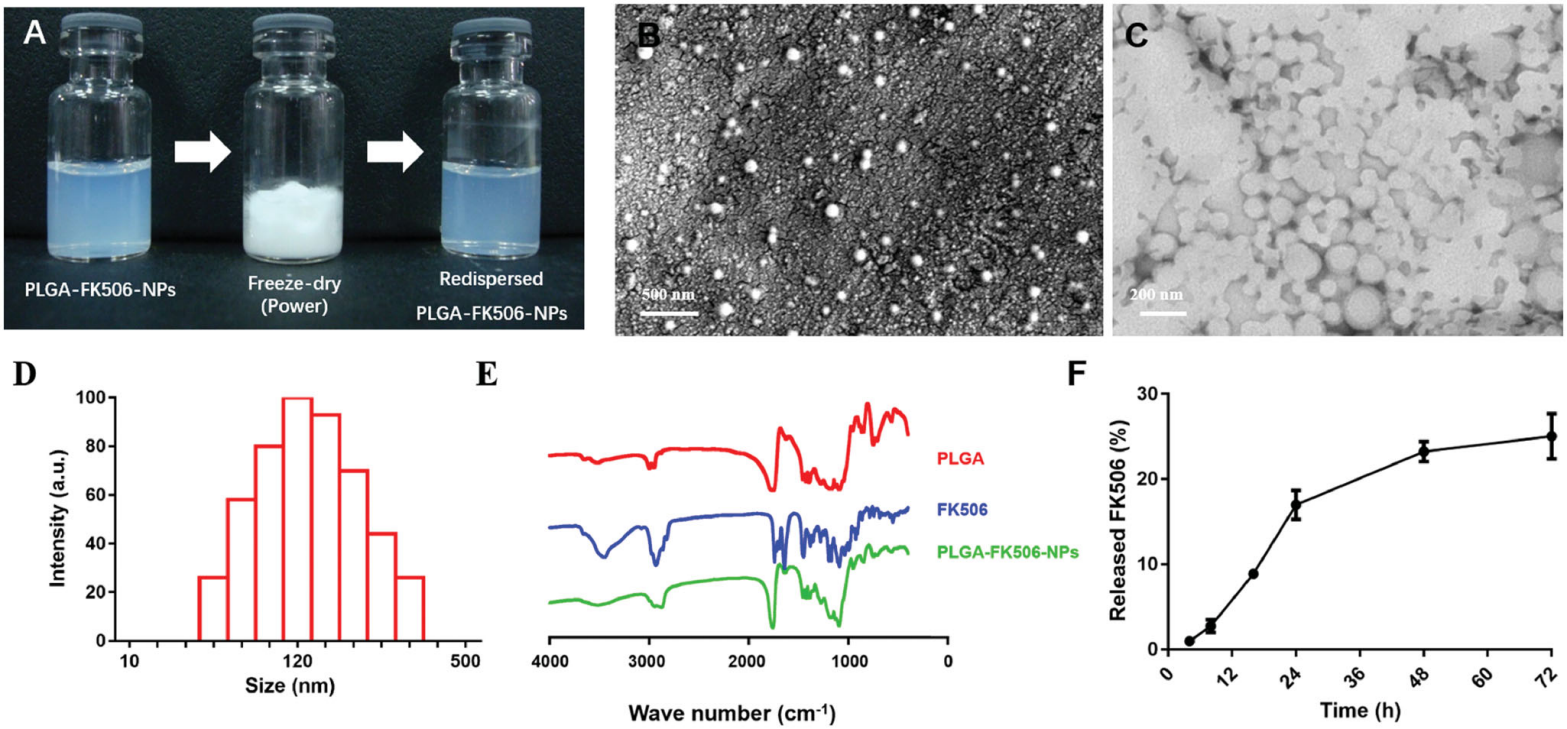
Characterization of PLGA-FK506-NPs. (A) Redispersibility of PLGA-FK506-NPs in PBS. (B) SEM and (C) TEM images of PLGA-FK506-NPs. (D) Size distribution and morphology. (E) FTIR spectra of PLGA, FK506, and PLGA-FK506-NPs. (F) *In vitro* release profile of PLGA-FK506-NPs in PBS at pH 7.4.

The PLGA-FK506-NPs were prepared by the O/W emulsion solvent evaporation method as previously reported (Deng et al., 2020). The dispersibility of PLGA-FK506-NPs is well maintained after lyophilization and redispersion, showing a unique opalescent color of nanoparticles (Figure 1(A)). The photos of PLGA-FK506-NPs under SEM and TEM reveal that they are monodispersed and spherical (Figure 1(B,C)). Consistently, the diameter of the PLGA-FK506-NPs is 122.5 ± 1.2 nm with a narrow size distribution (PDI = 0.149 ± 0.025) (Figure 1(D)). Furthermore, the zeta potential of the PLGA-FK506-NPs is −21.4 ± 1.76 mV. EE and EL of PLGA-FK506-NPs are 97.5 ± 1.13% and 5.74 ± 0.06%, respectively. The FTIR spectra of PLGA, FK506, and PLGA-FK506-NPs are shown in Figure 1(E). There was no difference in the spectra of PLGA and PLGA-FK506-NPs, which indicated the chemical-free encapsulation of FK506 in PLGA-NPs. The *in vitro* release profile of PLGA-FK506-NPs is shown in Figure 1(F). Approximately, 25.03 ± 2.17% of the FK506 was released within 72 h in a sustained manner. Note that DiR-labeled PLGA-NPs have an average diameter of 132.6 ± 2.0 nm and zeta potential of −17.43 ± 4.08 mV, which has properties similar to PLGA-FK506-NPs for the biodistribution study.

### 3.2. Biodistribution

**Figure 2.**
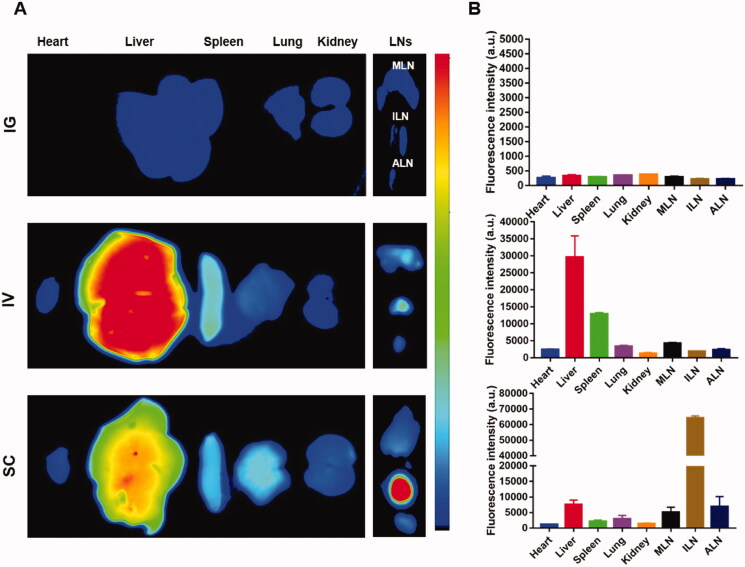
Biodistribution of DiR-labeled PLGA-NPs following various administration routes. (A) *Ex vivo* fluorescence image of major organs. (B) Quantitative analysis of NPs in major organs and LNs. MLNs: mesenteric lymph nodes; ILNs: inguinal lymph nodes; ALNs: axillary lymph nodes. Data are presented as the mean ± SD (*n* = 6).

Twenty-four hours post-injection of DiR-labeled PLGA-NPs via IG, IV, and SC, the major organs (heart, liver, spleen, lung, and kidney) and LNs (represented by MLNs, ILNs, and ALNs) were harvested for *ex vivo* fluorescence imaging. As shown in Figure 2(A), the fluorescence signal in LNs, especially in ILNs, was the highest among the observed organs in the SC group. In comparison, the fluorescence signals in the IV group are mainly accumulated in the liver and spleen. In the IG group, there is little fluorescence signal accumulated in the observed organs. The quantitative results in Figure 2(B) further demonstrated that the fluorescence intensity of ILNs was approximately 8.6 times higher than that of the liver in the SC group, demonstrating a high accumulation in LNs. In the IV group, the fluorescence intensity was mainly distributed in the liver and spleen, accounting for 50.4% of the total fluorescence intensity. Unexpectedly, no fluorescence intensity difference among the major organs was observed in the IG group.

### 3.3. Pharmacokinetic analysis

**Figure 3.**
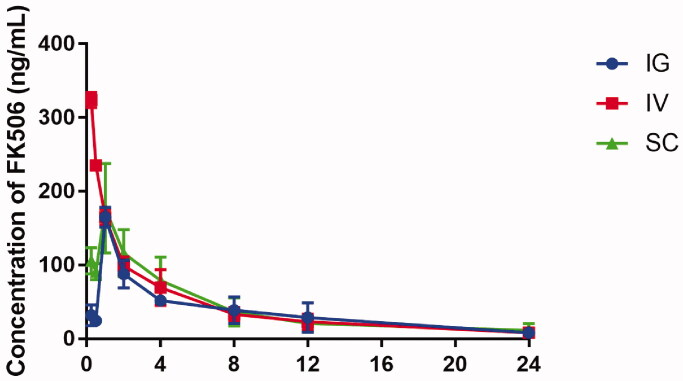
Blood concentration profiles of FK506 in normal rats after different administration of the PLGA-FK506-NPs. Each value represents the mean ± SD (*n* = 6).

**Figure 4.**
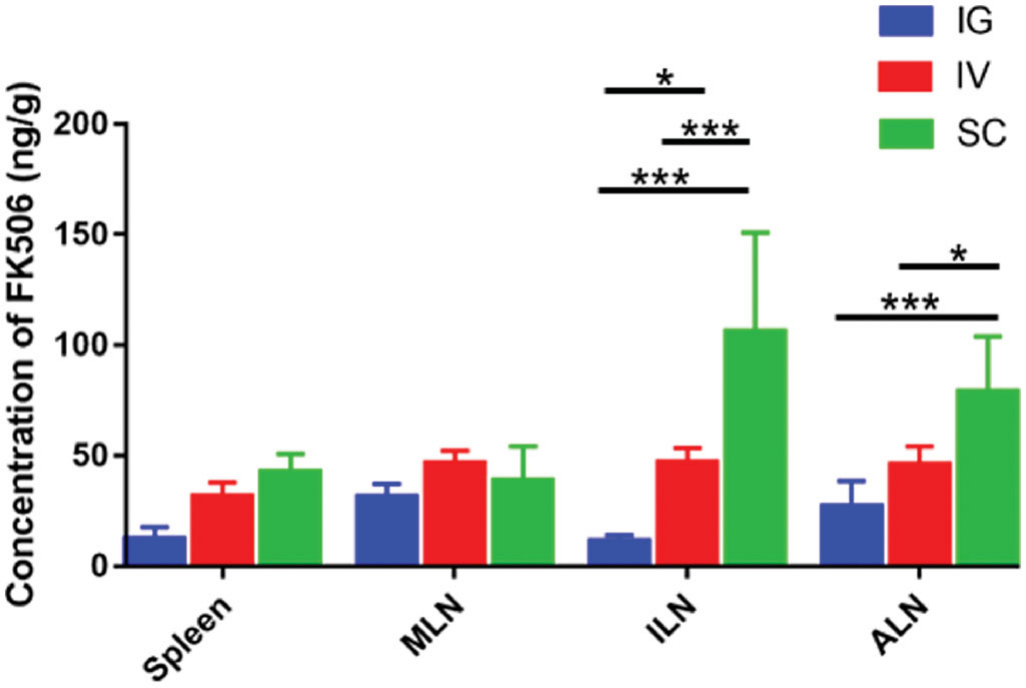
Lymphatic organs concentration profiles of FK506 in normal rats after different administration of the PLGA-FK506-NPs at 24 h. Each value represents the mean ± SD (*n* = 4). *p< .05, ***p< .001.

After demonstrating that the PLGA NPs can be accumulated successfully in LNs, the pharmacokinetics of PLGA-FK506-NPs with different administration routes were further evaluated by measuring the concentration of FK506 in whole blood, LNs, and spleen by using the HPLC–MS system at desired time points. The whole blood FK506 concentration–time curves of the three groups are shown in Figure 3. In IG group, FK506 was rapidly absorbed into circulation through the gastrointestinal tract with a maximum concentration (164.93 ± 13.40 ng/mL) at 1 h. Similarly, the drug was absorbed in the SC space, reaching a maximum concentration (186.14 ± 47.29 ng/mL) in SC group. Compared with SC or IG group, the maximum concentration was 323.76 ± 10.31 ng/mL in IV group and the FK506 concentration rapidly declined in circulation. Finally, all groups reach though concentration in 24 h after a single administration, and these results were similar to previous pharmacokinetic studies (Shin et al., 2010; Khan et al., 2016; Lee et al., 2019). The pharmacokinetic parameters are compiled in Table 1. The IV group had a significantly higher (1.23- or 1.15-fold higher) area under the whole blood concentration–time curve than IG and SC groups. The FK506 concentrations in the spleen and three LNs were also measured and shown in Figure 4. Notably, the FK506 concentration of ILNs and ALNs in the SC group resulted in a level of 105.86 ± 36.62 ng/mL and 78.88 ± 21.60 ng/mL at 24 h, while the FK506 levels were significantly lower in the IV group or IG group (46.76 ± 5.80 ng/mL, 45.74 ± 7.21 ng/mL and 11.27 ± 1.98 ng/mL, 26.68 ± 9.82 ng/mL), respectively.

**Table 1.** Pharmacokinetic parameters of FK506 after injection of PLGA-FK506-NPs in different administration routes.

Parameters	IG	IV	SC
*C*_max_ (ng/mL)	164.93 ± 13.40	323.76 ± 10.31	186.14 ± 47.29
*T*_max_ (h)	1	0.25	1
*t*_1/2α_	0.61 ± 0.40	0. 36 ± 0.09	1.2 ± 0.91
*t*_1/2β_	7.11 ± 2.14	4.35 ± 1.16	6.92 ± 2.02
AUC_0–_*_t_* (μg.h/L)	861.23 ± 232.15	1058.90 ± 225.98	920.95 ± 127.91
MRT_0–_*_t_* (h)	6.81 ± 1.54	5.32 ± 0.66	6.08 ± 1.34

*C*_max_: peak concentration; *T*_max_: peak time; *t*_1/2α_: distribution half-life; *t*_1/2β_: elimination half-life; AUC_0–_*_t_*: area under the whole blood concentration–time curve; MRT: mean residence time.

### 3.4. Histological and immunohistochemistry analysis

**Figure 5.**
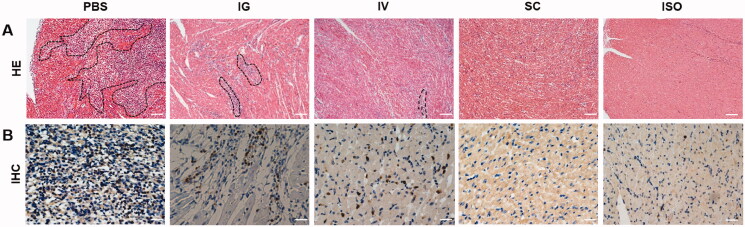
Histological and immunohistochemistry analysis of PLGA-FK506-NPs treatment via various administration on POD 7. (A) H&E staining of the tissue sections. Scale bar = 100 μm. (B) Immunohistochemistry staining with CD3+ T lymphocytes. Scale bar = 20 μm. Areas in black dotted lines indicate the areas of lymphocyte infiltration and myocyte damage.

To investigate the effects of different groups on the development of acute rejection-related pathology, we analyze the acute rejection grade in each group (*n* = 5) on POD 7. As shown in Figure 5(A) and Table 2, H&E staining revealed that the PBS group displayed massive lymphocyte infiltration, myocyte necrosis on POD 7, and 80% of samples exhibited grade 3R acute rejection. As expected, the ISO group, as the negative control, exhibited grade 0R rejection with no lymphocyte infiltration or myocyte damage. Treatment of PLGA-FK506-NPs in each route alleviates acute rejection to varying degrees compared with the PBS group. In more detail, we found that treatment with SC injection of PLGA-FK506-NPs significantly prolonged allograft survival compared with the other groups, and 80% of samples exhibited only grade 1R rejection on POD 7 with negligible lymphocyte infiltration. In contrast, the IV group exhibited only 40% grades 1R acute rejection with mild lymphocyte infiltration and 20% grade 3R that require further intervention, while the IG group exhibited grades 40% 3R acute rejection with more lymphocyte infiltration. The CD3 immunohistochemistry staining was further assessed to study the degree of T lymphocyte infiltration in the myocardium. The results are shown in Figure 5(B). Consistent with the above H&E findings, there was almost no CD3+ T lymphocyte infiltration in the SC group, and mild T lymphocyte infiltration was observed in the IG and IV groups. Many CD3+ T lymphocytes were infiltrated in the myocardium in the PBS group and almost no CD3+ T lymphocyte infiltration in the ISO group.

**Table 2.** Graft rejection grades in the groups after seven days.

Groups	Acute rejection grades			
	0R	1R	2R	3R
PBS			1	4
IG			3	2
IV		2	2	1
SC		4	1	
ISO	5			

### 3.5. Secretion of cytokines in cardiac allograft

**Figure 6.**
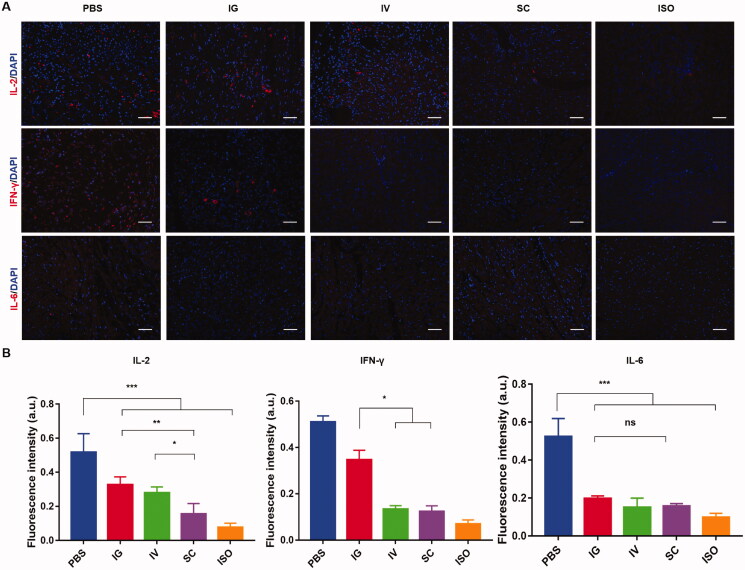
(A) Immunofluorescence staining of IL-2, IFN-γ, and IL-6 secretion in allografts or isograft. (B) Quantification analysis of fluorescence images. Scale bar = 50 μm. Each value represents the mean ± SD (*n* = 3). *p< 05, **p< .01, ***p< .001.

To investigate the effects of different groups on the secretion of cytokines, we further evaluated IL-2, IFN-γ, and IL-6 in cardiac allograft by immunofluorescence staining. The cytokines IL-2, IFN-γ, and IL-6 have been suggested as indicators of anti-graft immune response (Zheng et al., 2002; Jordan et al., 2017, 2020). Similarly, we found that IL-2, IFN-γ, and IL-6 were decreased in all treatment groups compared with the PBS group at POD 7 (Figure 6). The SC group showed a better result than the IV and IG groups for IL-2, IFN-γ, and IL-6. A similar conclusion can be drawn from the cytokine results as the above histological and immunohistochemistry have. The treatment with PLGA-FK506-NPs by SC administration inhibits the allograft rejection more effectively than the IG and IV routes.

### 3.6. PLGA-FK506-NPs SC administration prolongs cardiac transplant survival

**Figure 7.**
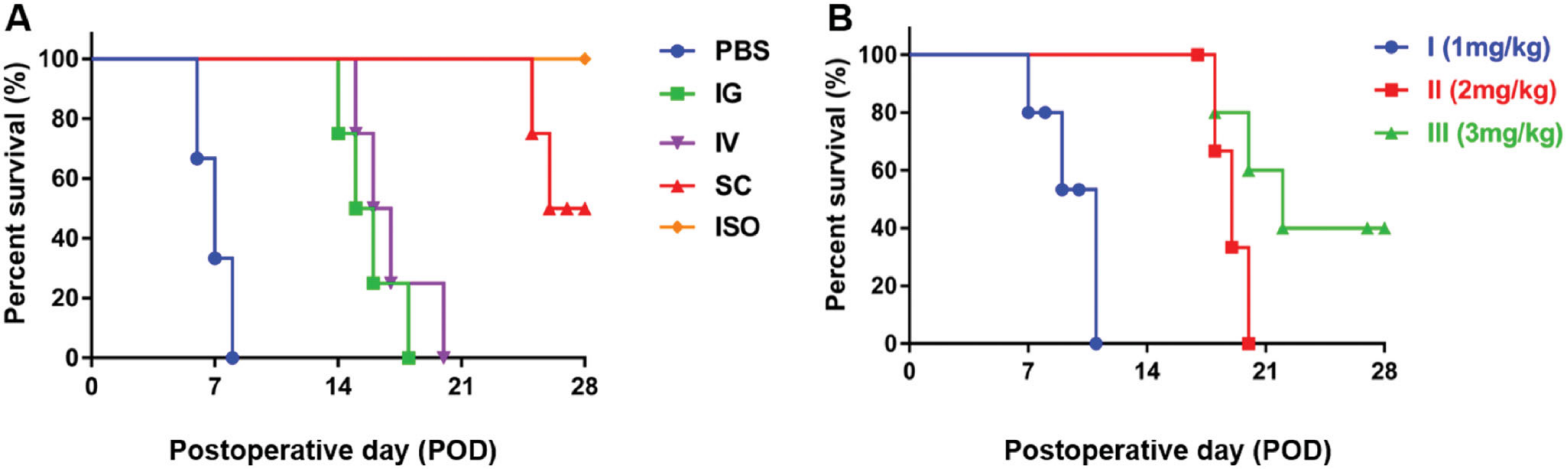
Kaplan–Meier’s survival curve of cardiac allograft treated with PLGA-FK506-NPs with different administration routes (A) and different doses via SC injection (B).

Finally, to evaluate the therapeutic efficacy of different routes on cardiac allograft, we compared the survival time of the grafts with different administration routes and the effects of different doses via SC injection. The Kaplan–Meier survival curve is shown in Figure 7(A,B). In addition, the group information and mean survival time (MST) are listed in Tables 3 and 4. Graft recipients given PBS rejected their grafts within about seven days. In contrast, cardiac grafts treated with IG or IV injection of PLGA-FK506-NPs survived within 21 days. Interestingly, three of six cardiac grafts treated with SC injection survived >28 days without any treatment beyond the POD 5. These results confirmed that the administration routes would strongly influence therapeutic efficacy, and SC was the most efficient route to deliver PLGAFK506-NPs to LNs to enhance the treatment effect. Moreover, the above-prolonged allograft survival was finely corroborated by histology analysis of the allografts at the end of observation (Fig. S1). Given the excellent feedback from the SC administration, we further studied the effects of PLGAFK506-NPs doses on the survival time via SC injection and the possibility of a single-dose cure by PLGAFK506-NPs SC injection. Graft recipients were given 1, 2, or 3 mg/kg single dose of PLGAFK506-NPs on the POD 1. As shown in Table 4, the graft survival time is prolonged as the dose increases. In detail, the 1 mg/kg dose slightly increases the MST compared with the PBS group (8.7 vs. 7.0 days; *p* < .05). In the 2 mg/kg group, the MST effectively extends to 19.2 days. Finally, the 3 mg/kg single dose made two in five of the cardiac grafts survived >28 days, which is not effective enough as the above five consecutive doses are.

**Table 3.** Graft survival time of heterotopic abdominal cardiac transplantation rats treated with different routes of administration.

Groups	*N*	Mean survival time (days)
PBS	6	7
IG	5	16.2*
IV	6	17*
SC	6	>26.8^§^
ISO	4	>28^#^

Administered doses of FK506 were 1 mg/kg, five times. Graft survival time plotted at >28 means that the graft survived more than 28 days.

* *p*< .05 vs. PBS.

§ *p*< .01 vs. PBS, IG, IV.

# *p*< .05 vs. PBS, IG, IV.

**Table 4.** Graft survival time of heterotopic abdominal cardiac transplantation rats treated with subcutaneous administration.

Group	Dose	*N*	Mean survival time (days)
I	1 mg/kg once	7	8.7
II	2 mg/kg once	4	19.2
III	3 mg/kg once	5	>23.2

## 4. Discussion

Currently, FK506 is one of the most commonly used immunosuppressants in HT. Due to its limitations, including narrow therapeutic window, poor solubility, and low bioavailability via oral administration, long-term use of FK506 needs to balance adverse effects and therapeutic efficiency in a clinical regimen. Hence, a change in the route of drug administration would be a powerful strategy to overcome its limitations (Solari et al., 2017; Gao et al., 2021). According to the literature, SC administration is a popular alternative delivery route as it provides the following advantages, such as (1) potential to avoid enteral metabolism and liver first-pass effect and to promote the effective delivery of drugs that have low oral bioavailability; (2) potential to achieve improved tolerability by minimizing the peak to trough ratio in the systemic circulation; (3) potential to achieve prolonged systemic exposure (Chiang et al., 2019; Lee et al., 2019). Therefore, it is necessary to verify whether different administration of immunosuppression influences outcomes.

In this study, we investigated PLGA-FK506-NPs with different routes of administration to treat cardiac allograft acute rejection, and we characterized the pharmacokinetics of PLGA-FK506-NPs after IG, IV, and SC administration in rats. The results suggest that SC injection as an alternative dosing route prolonged the survival time and reduced the grade of acute rejection, consistent with other related studies performed on transplantation. It has been reported that SC injection of FK506-loaded microspheres to treat rejection in rat liver transplantation model (Miyamoto et al., 2004). It maintained a stable blood concentration for about 10 days and significantly prolonged the graft survival time. In another study, an enzyme-responsive FK506-loaded hydrogel was SC injected in a vascularized composite allograft model, which prolonged the survival time and reduced drug dosage simultaneously. Most importantly, the SC route reduced the systemic drug concentration and avoided the incidence of adverse effects (Gajanayake et al., 2014). The advantages of reduced dose combined with the sustained release behavior could make the SC injection be a new strategy for administering FK506.

The results of the pharmacokinetics study showed that PLGA-FK506-NPs with different routes of administration have similar trough concentrations. However, the concentration of FK506 in ALNs and ILNs in the SC group was much higher than in the IV and IG groups. In addition, it has been confirmed that LNs is critical for acute rejection and targeted delivery of drug have the potential to improve the efficacy (Bahmani et al., 2018; Zhou et al., 2021). Although the specific changes of immunocyte activation, proliferation, and secretion of cytokines in LNs were not investigated in detail, to some extent, the prolonged survival time may be attributed to the higher local concentration of FK506 in LNs, which efficiently inhibited the priming and activation of T lymphocytes.

Consistent with the results of the biodistribution study in the previous study (Rao et al., 2010), NPs mainly accumulated in ALNs in the SC group, and its fluorescence intensity was much higher compared with the IV and IG groups, while the NPs mainly accumulated in the liver and spleen in IV group. Our findings indicate that the routes of administration influence the distribution of NPs. It is well known that the distribution of NPs after IV injection is located at the sites of the mononuclear phagocytic system, such as the liver, spleen (Liu et al., 2008). The SC injection is different, based on the SC tissue contains abundant capillaries and a special structure of capillary lymphatic vessels. Most of the small molecule drugs are absorbed by the capillary. Since the diameter and permeability of capillary lymphatic vessels are much larger than the capillaries, the absorption of the nanoparticles is different from small molecule drugs, which largely depends on the size of nanoparticles. Nanoparticles less than 10 nm in diameter are directly absorbed by the capillary, while nanoparticles of 10–100 nm are preferentially absorbed by lymphatic capillaries, enter LNs and then circulate through lymph vessels to the whole body (Reddy et al., 2006; Trevaskis et al., 2015). In comparison, nanoparticles with a size larger than 100 nm enter local LNs through phagocytosis and circulate through lymph vessels to the whole body (Jiang et al., 2017). In this way, drugs can interact with immune cells in the LNs, regulating the response of the immune cells.

Survival time is the key index to evaluate the therapeutic effect. The result in Figure 7 verified that SC injection of PLGA-FK506-NPs was the best among all the groups. Fifty percent of allograft survival over 28 days. Compared with the IV or IG group, a single SC injection of PLGA-FK506-NPs (3 mg/kg) almost reached the same efficiency, resulting in 40% of allograft survival over 28 days. Recently, with the development of a long-term drug release system via SC administration of immunosuppressive drugs, an MST of more than 150 days was archived in a rat hind limb transplantation model (Wang et al., 2020). Moreover, T lymphocytes, which play a critical role in acute rejection, are associated with the degree of myocardial injury (Pietra et al., 2000). Histological examination of heart grafts displayed in Figure 5 showed evidence of acute rejection and lymphocyte infiltration as well as myocyte injury in the IV or IG group, while the situations were much better in the SC group. It is well known that reducing the secretion of IL-2 and IFN-γ would significantly decrease the total number of T lymphocytes and eventually alleviate acute rejection. Moreover, the pro-inflammatory cytokine IL-6 also plays a pathological effect on immune response, and it has been confirmed that IL-6 mediates numerous immunologic effects relevant to transplant rejection (Booth et al., 2011; Tanaka et al., 2014). As shown in Figure 6, treatment with PLGA-FK506-NPs via SC injection inhibits the secretion of cytokines compared with other groups. Taken together, these results indicate that SC injection of FK506 could effectively suppress acute rejection and prolong survival.

## 5. Conclusions

In this study, we characterized PLGA-FK506-NPs *in vitro* and *in vivo*. The pharmacokinetics of PLGA-FK506-NPs following SC, IV, or IG administration were compared in rats. Finally, the therapeutic efficacy of PLGA-FK506-NPs using different routes of administration was compared in a HT model. In all, these results suggest that SC injection can be an alternative administration route for systemic delivery of FK506 in animal models of transplantation. More importantly, our findings demonstrated that SC administration may be considered for short-term use in patients who cannot receive medications orally. This new method could be used to eliminate daily systemic drug administration, improve patient compliance, reduce drug dosage, and minimize toxicity and complications in the future.

## Supplementary Material

Supplemental Material
