# Supplementary material for: Immunosuppressive effect of PLGA-FK506-NPs in treatment of acute cardiac rejection via topical subcutaneous injection
Source: Drug Deliv. 2021 Aug 31;28(1):1759–68. doi: 10.1080/10717544.2021.1968978 (PMC8409942; doi:10.1080/10717544.2021.1968978)
Supplement: Supplemental Material [file IDRD_A_1968978_SM6102.docx]

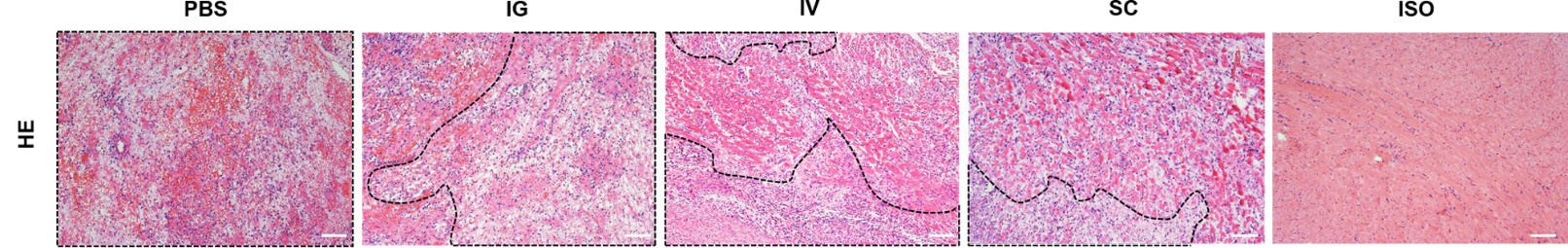


**Figure. S1.** Histology analysis of the allografts at the time of complete cessation of heart beats among different groups. Areas in black dotted lines indicate the areas of lymphocyte infiltration and myocyte damage.
